# Narratives, masks and COVID-19: A qualitative reflection

**DOI:** 10.1177/1473325020973330

**Published:** 2021-03

**Authors:** Jade Wong, Emily Claypool

**Affiliations:** School of Social Service Administration, University of Chicago, Chicago, USA

**Keywords:** Narrative, power, discourse, critical social work

## Abstract

Tracing explanatory narratives of mask-wearing throughout COVID-19, we argue that multiple narratives contribute to the global experience of COVID-19, making it as much a social and political object as it is a scientific one. This assumption drives our commitment to take seriously alternative narratives that do not conform to dominant ones in order to examine how structures of power might privilege particular types of ‘truths’ and with what consequences. We see this reflective piece as a re-articulation of social work’s historic call to interrogate dominant ways of knowing, particularly the ways in which science obscures its own power and politics and sidelines other narratives in the process.

We might be tempted to view COVID-19 in dramaturgical terms: It “start[s] at a moment in time, proceed[s] on a stage limited in space and duration, following a plot line of increasing and revelatory tension, move to a crisis of individual and collective character, then drift toward closure” (Rosenberg, 1992: 279). And so, we wait with bated breath for the winnowing of plots of uncertainty and crises for when the truth of the matter is revealed – culprits are exposed, heroes are celebrated and retrospective accounts, told. In the meanwhile, we are confronted with a thoroughly postmodern disease that refuses to be contained to a single plot line. What is its cause? Is it the bats, rats, or ‘bad’ hygiene? Who are the culprits? The U.S. government and its laggardly response, the CDC or WHO that should have known better, or perhaps everyday civilians who refuse to wear masks and wash their hands?

Despite the cacophony of voices that uniformly suggests the pandemic may evade a single, or even a few, plotlines, what is remarkable about this moment in time is our insistence to find a single explanatory narrative to make sense of it. Yet, because the temptation to retell its dominant narratives swells as the story of COVID-19 seemingly “drift[s] to closure,” our call is to take seriously the putatively irrational, peripheral, or nonscientific narratives that crop up. Social work has long been aware that narratives are political because they are imbricated in structures of power that produce hierarchically-stratified truths in given moments (Hartman, 1990). We therefore consider attachments to select narratives as consequential for they provide apparatuses of sensemaking and modes of possible intervention. After all, narratives can be “just as real as germs”, with “real effects on how people live and die” (Briggs and Mantini-Briggs, 2002: 7).

To illustrate the stakes involved in the production and transmission of narratives, we trace explanatory narratives of mask wearing – that thin, often-pleated, material we lay on top of our mouths and noses – throughout COVID-19 to examine which narratives appear to glide to the top more smoothly than others as well as those which tend to get sidelined.

## The narrativity of face masks

**Figure 1.**
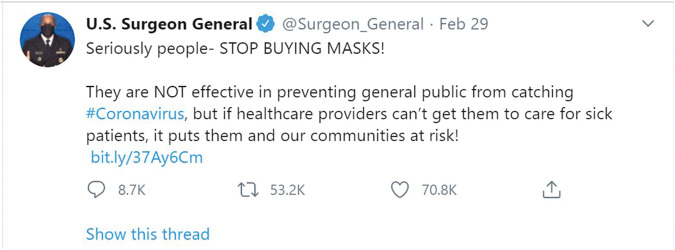
Dr. Adam Jerome’s Tweet.

In the months following WHO’s declaration that COVID-19 is a pandemic, the CDC and U.S. public health experts advised the public against wearing face masks unless they have COVID-19 or care for people who do. Denouncing the masks’ ability to protect, the U.S. Surgeon General, Dr. Jerome Adams, demanded the American public to “STOP BUYING MASKS!” (See Figure 1).

Yet, with the scientific discovery of the virus’ potential for airborne flight from people without symptoms (“asymptomatic”) or at the cusp of developing symptoms (“pre-symptomatic”) to unknowing bystanders, the CDC reversed its recommendation on April 3^rd^. The CDC refurbished its website with a dedicated section on cloth coverings, declaring in the text: “In light of this new evidence, CDC recommends wearing cloth face coverings in public settings.” The text is accompanied by a 45-second video of Dr. Adams, who previously tweeted “STOP BUYING MASKS,” refashioning a black t-shirt harkening back to another epidemic, “got naloxone?”, into a face covering (Centers for Disease Control and Prevention, 2020).

**Figure 2.**
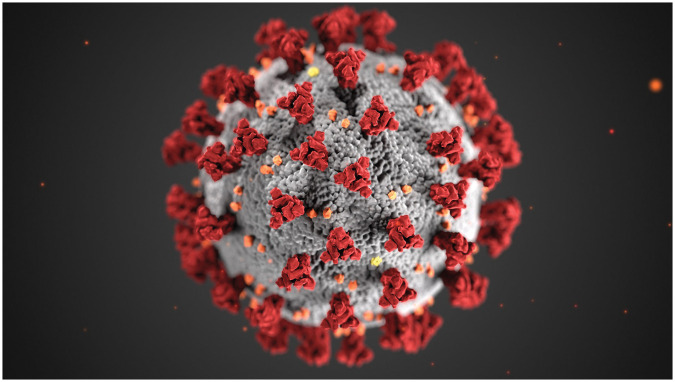
3-D rendering of COVID-19.

Insulated by an ideology of scientific progress, there is rarely culpability in science for the provision of suspect advice. Instead, there is simply scientific uncertainty caused by the wily of a virus whose chaos evades scientific capture. Consider, for example, Figure 2 which shows the CDC’s portrayal of the “identity” of the virus (Giaimo, 2020). Historian of medicine, Michael Rossi, describes this “beauty shot” – a 3-D floating blob adorned with spikes – as stirring an image that “somewhere, a coalition of scientists, administrators and elected officials are working on the problem” (Rossi, 2020), luring us to interpret the outpouring of advice as a victorious sign of scientific progress while issues such as the highly racialized effects of the disease dwindle in significance.

But could the experts really not anticipate the importance of face masks and coverings? After all, since September 11 and Hurricane Katrina, institutions of “Emergency Preparedness” have proliferated in American bureaucratic life, readying workers for anticipatory action through the arcane and mundane practices of safety drills and online trainings (Murthy et al., 2017). As early as January, everyday individuals in Hong Kong, South Korea, and Taiwan donned the mask while American experts centered their efforts on extolling the virtues of washing your hands for 20 seconds and staying 6 feet apart (Jingnan, 2020).

In an interview in mid-June, Dr. Anthony Fauci, Director of the National Institute of Allergy and Infectious Diseases and America’s poster child for ‘sticking to the scientific facts’ amid COVID-19, conceded the public was not advised to wear masks due to the national shortage in personal protective equipment (Ross, 2020). Writing in the *New Yorker*, Mukherjee (2020) learned that the Strategic National Stockpile, which deploys emergency equipment on behalf of the federal government on short notice, only had 13 million N95 masks on hand. He notes that if a fifth of the workforce in New York and California had contact with positive-tested patients and burned through two N95 masks per worker per day, the stock would last a mere eleven days. Calling medicine “a complex web of systems and processes” (p. 24) Mukherjee laments “[t]he shortage of these mass-manufactured fifty-cent items [masks] have imperilled the safety of our medical personnel” (p. 25).

Yet the shortage of personal protective equipment does not change the fact that masks were deemed non-essential for months. Skirting the limits of truth through the play of words, the CDC, Dr. Fauci, among others drew a line between “masks” and “coverings”. They reminded their adherents that coverings are distinct from surgical masks and N-95 respirators, and that masks ought to be conserved for healthcare workers. Furthermore, they assured the public that their previous guidance was offered when the facts of airborne spread were not yet determined, however now “we know that you don’t need an N95 if you’re an ordinary person in the street,” and “simple cloth coverings that many people have can work as well as masks in many cases” (Ross, 2020).

On the same day that the CDC revised its recommendations, the U.S. Health and Human Services Secretary, Alex Azar, declared his allegiance to face coverings. Appealing to “the science” which “suggests it will help prevent you from spreading it to others,” he cajoled, “face coverings are a new, voluntary opportunity for a unified national effort […]. Taking this step will protect our loved ones, our communities, and our country from the invisible enemy we’re fighting together” (Department of Health & Human Services, 2020). Through invoking the credibility of science and rhetoric of individual responsibility, everyday individuals become entangled in the narrative of face coverings. We can choose to be heroes by sewing makeshift coverings from recycled fabric in a collective endeavor bolstered by national pride to wage war against the “invisible enemy”. Or we can ignore this call, just like President Trump who expressed his preference to not follow suit (Bloomberg News, 2020).

For some skeptics, including Dr. Judy Mikovits in her 26-minute documentary titled *Plandemic* on the causes of COVID-19, donning masks carries serious health concerns. She warned that flu vaccines implant a dormant form of coronavirus into peoples’ bodies, which will “literally activate” upon wearing masks. Despite *Plandemic’s* expulsion from social media platforms such as YouTube, Facebook, Vimeo and Twitter, it remained one of the most widely circulated repositories of conspiracy theories on COVID-19 (McGreal, 2020). We might be so inclined to call Dr. Mikovits and her adherents quacks before casting them aside or ‘sympathetically’ rendering them, as a New York Times reporter did, as “people whose critical faculties have simply been overwhelmed […] by feelings of confusion and helplessness” (Fisher, 2020). But what if we shift our eyes from the believers’ mental states and listen to what they say? Perhaps the refusal to wear masks spoke to concerns of government’s heavy-handed involvement in individuals’ health as well as the perceived regulatory capture of the government by Big Pharma. Or, perhaps it channeled skepticism over whether medical progress ultimately leads to the flourishing of life or the curtailment of autonomy over our own bodies.

Fierce individualism is intertwined with American Folklore, which is evidenced in their right to bear arms, protest, and refuse the “voluntary opportunity” to wear face coverings. On May 1^st^, the owner of a grocery shop in Manhattan’s Chinatown asked two customers to wear masks, but the customers refused. This escalated to an argument, police intervention, and the arrest of all parties involved. At the same time, anti-lockdown protests broke out in many states across the United States, with participants airing rifles and handguns without donning masks (DeBrabander, 2020). This elicited the question among some commentators: do masks have less to do with science than ‘culture’ (Jingnan, 2020)? After all, noting that people from Asian countries seem more at ease with wearing masks, it could be crudely presumed that belonging to an ‘authoritarian’ regime makes one more amenable to wearing masks (Jingnan, 2020).

Running parallel to the narratives of COVID-19 were the murders of Black lives – Ahmaud Arbery, Breonna Taylor, Dion Johnson, George Floyd, Tony McDade, among others. As protests erupted worldwide, the face mask, an icon of the fight against a virus that attacks the respiratory tract, was written in bold black lettering to reflect George Floyd’s words, “I can’t breathe,” so as to echo the experiences of asphyxiation and silence felt by Black lives (Crawford, 2020). Engaging in protests may seem unfathomable given they are “super-spreading” events, putting individuals in harm’s way as respiratory droplets might burst forth from the infected person’s mouth when they chant a slogan, sneeze at the scent of pepper spray, or cough when they inhale tear gas. Yet invoking the trope that COVID-care is the responsibility and agency of individuals, one protestor told an NBC reporter, “I can go home, clean myself up, get tested, make sure I take proper precautions. But police brutality, I don’t know,” for there is a world of difference between a virus that presumably can be controlled through personal hygiene and a system of police brutality and racial violence that escapes it (Wolfe, 2020).

## Reflections

The incoherence, messiness and radical uncertainty that characterizes COVID-19 is marked by an intense moment of observation and scouring for information, oftentimes competing and only partial. Any anxious or inquisitive individual is easily confronted with a deluge of models, speculations, and Twitter anecdotes about, for example, the manner by which the disease spreads, the demographic of individuals it tends to affect more acutely, and the types of drugs or protocols that may stymie its effects. While COVID-19 has revealed the differential health effects arising from immense and long-standing racial and economic inequality, our concern is that the response has been largely one of invoking individual responsibility such as washing hands, wearing masks, and social distancing. This is reminiscent of Briggs and Mantini-Briggs’ (2002) concept of *sanitary citizenship*, where the ‘public’ in public health is envisioned and thus interpellated as prudent and responsible by adopting particular hygiene practices, rendering those who are perceived as incapable of such practices as “unsanitary” or, in the case of COVID-19, scientifically naïve and/or socially irresponsible.

As we have shown, even narratives concerning face masks, including shortages in personal protective equipment, semantic quibbles over masks versus coverings, conspiracy theories, police shootings and protests, run alongside, interweave and intersect with COVID-19. The multitude of narratives and voices in many ways capture the essence of COVID-19 that would otherwise be glossed over in neat timelines and stylized histories. These narratives, along with many more, accrete and interact to contribute to the global experience of COVID-19, making it as much a social and political object as it is a scientific one (Lindenbaum, 2001). The experience of COVID-19 therefore reminds us to view this presumably scientific object as the synergy of biological, social and political conditions that contribute to and bring forth the unequal burden of disease and social ills. It reminds us to pay heed to social work’s historic call to interrogate dominant ways of knowing, taking seriously those narratives that do not conform to prevailing ones in order to recognize the depth and complexity of the disease and its effects (Staller, 2016). Finally, we are reminded to examine the structures of power that produce, highlight, and silence particular ‘truths’ in given moments, lest we allow certain narratives mask the multiplicity of experiences that make up COVID-19.
